# Oculomotor abnormalities in patients with cerebellar ataxia, neuropathy, vestibular areflexia syndrome (CANVAS) reflect midline cerebellar impairment

**DOI:** 10.1007/s00415-026-13678-4

**Published:** 2026-02-14

**Authors:** Renana Storm, Bente Lübbers, Max Borsche, Emmelie Weiss, Astrid Nümann, Christos Ganos, Norbert Brüggemann, Christoph Helmchen, Andreas Sprenger

**Affiliations:** 1 Department of Neurology, University Medical Center Schleswig-Holstein, Campus Lübeck, Lübeck, Germany; 2 Center of Brain, Behavior and Metabolism (CBBM), University of Lübeck, Marie-Curie-Straße, 23562 Lübeck, Germany; 3 Institute of Neurogenetics, University of Lübeck, Lübeck, Germany; 4 Department of Neurology With Experimental Neurology, Charité-Universitätsmedizin Berlin, Corporate Member of Freie Universität Berlin and Humboldt-Universität Zu Berlin, Berlin, Germany; 5 Experimental and Clinical Research Center (ECRC), Charité-Universitätsmedizin Berlin, Berlin, Germany; 6 Edmond J Safra Program in Parkinson’s Disease, Morton and Gloria Shulman Movement Disorders Clinic, Toronto Western Hospital, University Health Network, Toronto, ON Canada; 7 Division of Neurology, University of Toronto, Toronto, ON Canada; 8 Institute of Psychology, University of Lübeck, Lübeck, Germany

**Keywords:** CANVAS, *RFC1*, Oculomotor, Saccades, Nystagmus

## Abstract

**Background:**

Biallelic intronic repeat expansions in the *RFC1* gene are associated with the cerebellar ataxia, neuropathy, and vestibular areflexia (bilateral vestibulopathy) syndrome (CANVAS). Oculomotor abnormalities may serve as a state marker exclusively reflecting the midline cerebellar involvement in CANVAS, i.e., independent of the combined disorders affecting the patient’s postural control stance and gait.

**Methods:**

Slow and fast eye movements of 15 CANVAS patients and 14 healthy subjects were compared using a high-resolution video-based eye tracker allowing to record visually-guided saccades, gaze-holding function and smooth pursuit paradigms. Scores of cognitive impairment were related to oculomotor performance.

**Results:**

Saccades (latency, metria, velocity) were normal. Small amplitude omnidirectional gaze-holding deficit was found in 70% of patients, with downbeat nystagmus (60%) being more common than upbeat nystagmus (13%). Latency of initial acceleration of smooth pursuit was prolonged and there was severe impairment of smooth pursuit eye movements. Montreal Cognitive Assessment (MoCA) scores were lower in patients and correlated with saccade and pursuit latency and initial acceleration. Disease duration and vestibulopathy correlated with no oculomotor abnormalities.

**Conclusion:**

Cerebellar oculomotor dysfunction affected mainly smooth pursuit and gaze holding function at eccentric gaze positions, but it did neither comprise spontaneous nystagmus nor saccade abnormalities. Prolonged latencies in initial pursuit acceleration might be related to the patient’s cognitive decline, but normal saccade latencies point to cerebellar oculomotor neurodegeneration. Smooth pursuit impairment was not related to disease duration and vestibulopathy, contrariwise pursuit impairment became worse with larger functional impairment. Oculomotor abnormalities in CANVAS are in line with midline cerebellar impairment without evidence for extracerebellar (brainstem) brain involvement.

**Supplementary Information:**

The online version contains supplementary material available at 10.1007/s00415-026-13678-4.

## Introduction

The combination of **c**erebellar **a**taxia, **n**europathy, and **v**estibular **a**reflexia as CANVAS (**s**yndrome) is pathognomonic for the biallelic intronic repeat expansion in the *RFC1* gene [1], usually preceded by decades of chronic dry cough. Ever since, progression of sensory and bilateral vestibulopathy due to ganglionopathy has been described in detail [2, 3] but the risk of falls increases rapidly with additional cerebellar involvement, dysautonomia and cognitive decline [4]. As ataxia of gait and stance may be related to any of the impaired systems, oculomotor abnormalities may serve as a state marker exclusively reflecting midline cerebellar involvement in CANVAS, i.e., independent of the combined disorders affecting the patient’s postural control and gait. Cerebellar oculomotor control is mainly governed by the midline cerebellum, i.e., vestibulocerebellum [5]. The suppression of the vestibulo-ocular reflex (VOR) is clinically applied to identify cerebellar impairment but may be misleading, i.e., false negative, in CANVAS patients. The patient maintains fixation during head/chair rotation due to the subtotal loss of vestibular function (vestibular ganglionopathy). Accordingly, there is no need to elicit smooth pursuit eye movements to suppress the VOR. Therefore, it is recommended to use the visually enhanced vestibulo-ocular reflex (VVOR), which requires smooth pursuit eye movements to maintain fixation of an earth-fixed target at the gaze straight ahead position. The saccadic instead of smooth pursuing eye movements impressively demask midline cerebellar impairment [6, 7]. Recently, slow eye movements were analyzed in a large CANVAS cohort, indicating that the impairment of the VVOR gain and smooth pursuit eye movements were related to the length of the *RFC1* intronic expansion size as the deficits increased with the expansion length [8]. For technical reasons (low resolution: 60 Hz frequency), however, fast eye movements could not be assessed. Here, we investigated *fast* eye movements in CANVAS to broaden the oculomotor phenotype, i.e., saccades, gaze holding function, and initial acceleration of smooth pursuit in 15 patients with confirmed biallelic *RFC1* repeat expansions. Based on the assumption of vestibule-cerebellar neurodegeneration, we hypothesized to find saccade dysmetria with normal saccade velocity, impaired initial pursuit acceleration and gaze-holding impairment.

## Methods

### Participants

The group of CANVAS patients comprised 15 carriers of biallelic repeat expansions in the *RFC1* gene enrolled in this prospective study at the Department of Neurology, University Hospital Schleswig–Holstein, Campus Lübeck, Lübeck, Germany. Their data were compared to 14 likewise prospectively investigated age-matched healthy control subjects (HC). The study was approved by the local Ethics committee of the University of Lübeck (AZ 20–358). Written informed consent was obtained from all participants. Genetic testing of biallelic *RFC1* repeat expansion was performed at the Institute of Neurogenetics, University of Lübeck, Lübeck, Germany, as described [9], by a screening step performing duplex PCR analyses, repeat-primed PCR, and Sanger sequencing.

Disease onset was defined by patients reporting to have recognized symptoms associated with vestibular or cerebellar dysfunction, or neuropathy such as stand, gait or speech disturbance, limb ataxia, or oculomotor abnormalities (gaze-evoked blurred vision or diplopia, oscillopsia) for the first time. The Scale of the Assessment and Rating of Ataxia (SARA) was used to quantify ataxia, and the Montreal Cognitive Assessment (MoCA) to determine cognitive impairment (Table 1). Healthy participants were excluded with MoCA values below 25 and SARA scores above 2 [10]. All participants underwent a neurological examination, including static and dynamic subjective visual vertical (SVV) and quantitative analysis of the gain of the vestibulo-ocular reflex (VOR) via video-based head impulse test (HIT, Table 1). Details on vestibular recordings are described in the supplementary information. Fampridine was taken by three patients at the time of examination. No other centrally acting medication was used by patients or HC.

**Table 1:** Demographics and clinical scores of participants

	CANVAS (mean ± STD)	HC (mean ± STD)	Statistical difference
Number	15	14	n/a
Age	65.40 ± 9.92	65.36 ± 7.40	*p* = 0.880
Age at onset	52.60 ± 9.19	n/a	n/a
Disease duration (years)	12.40 ± 6.55	n/a	n/a
MOCA	24.73 ± 3.81	27.36 ± 3.08	***p* = 0.036**
SARA	12.20 ± 5.09	0.39 ± 0.70	***p* < 0.001**
VOR Gain	0.15 ± 0.13	1.01 ± 0.15	***p* < 0.001**
SVV stat	3.47 ± 2.83	1.33 ± 0.96	***p* = 0.041**
SVV dyn	4.88 ± 3.31	1.71 ± 1.11	***p* = 0.007**

Significant differences are marked bold

CANVAS = Cerebellar Ataxia with Neuropathy and Vestibular Areflexia Syndrome, HC = healthy control subjects, MOCA = Montreal Cognitive Assessment, n/a = not applicable, SARA = Scale for the Assessment and Rating of Ataxia, SVV stat. = static subjective visual vertical, SVV dyn. = dynamic subjective visual vertical, VOR = vestibulo-ocular reflex

### Oculomotor recordings

Eye movements were recorded with two video-based eyetracking systems: half of the subjects were recorded with the head-mounted Eyelink II system (native sampling rate 500 Hz) and the other half of subjects by the Eyelink 1000 Plus (native sampling rate 1000 Hz; both systems from SR Research Ltd., Ontario, Canada). Details on the exact experimental setup of stimuli as well as the recording program can be found in the supplementary information and previous studies [11, 12]. We investigated all participants (patients and HC) under head-stationary conditions in the dark using the following paradigms which are described in details in the supplementary information and elsewhere [12]: reflexive horizontal and vertical saccades (amplitudes: 5°, 10°, 15°), fixation at gaze straight ahead and on horizontal and vertical eccentric gaze positions (hor: 20°, ver: 10°) as well as smooth pursuit paradigms (sinusoidal and step-ramp).

### Data and statistical analysis

Eye movement data were analyzed using custom-made software in Matlab^®^ (R2024b, The Mathworks Inc., Natick, MA, USA). Saccade and blink detection were checked interactively. Each trial of the different tasks was manually verified. Eye movement performance was calculated as gain values for saccade tasks (eye amplitude/target amplitude) and smooth pursuit tasks (eye velocity/target velocity). Saccade peak velocity was derived from a main sequence fit procedure using power-to-fit-function within Matlab^®^ and calculated for 10° and 15° amplitudes. Nystagmus during gaze holding function was quantified via slow phase velocity (°/s) and analyzed separately for direction (horizontal, vertical).

Normality test failed for all analyses. Subsequently, we used non-parametric tests (Mann–Whitney-U test) for analyzing group differences and Spearman’s Rho for correlations. Results are stated as mean values with standard deviation (SD). Statistical differences were regarded as significant for values *p* < 0.05. Box plots show the median (midline) and a box (25th to 75th percentile of the distribution). Error bars indicate the minimum/maximum value or 1.5*interquartile range if outliers (circles) are present.

## Results

We investigated 15 *RFC1*-positive patients (4 female) with the complete clinical presentation of the triad of bilateral vestibulopathy, cerebellar ataxia, and neuropathy at the time of study enrollment (Table 1).

There was no main effect of target direction in any task nor an interaction between target direction and group; therefore, data were averaged for direction.

**Fig. 1:**
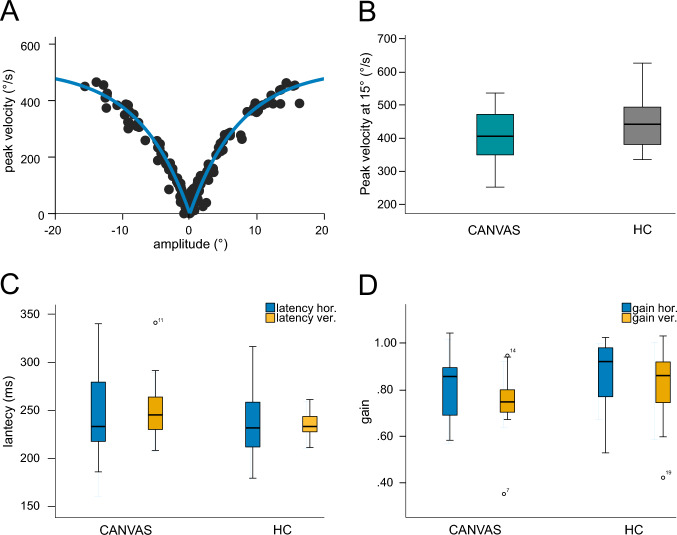
Saccades: peak velocity of horizontal saccades as main sequence fit for one patient (**A**). There are no group differences of saccade peak velocity at 15° (**B**). Latency (**C**) and gain (**D**) for horizontal and vertical saccades between patients and HC. None of the saccadic parameters differed between groups, i.e. patients showed preserved saccadic function. Abbreviation: CANVAS = cerebellar ataxia, neuropathy, vestibular areflexia syndrome, HC = healthy control subject

*Saccades*: For saccades, there were no group differences (*p* always > 0.108) with respect to horizontal and vertical saccade amplitude gain (horizontal, CANVAS: 0.82 ± 0.13, HC: 0.88 ± 0.17; vertical, CANVAS: 0.72 ± 0.04, HC: 0.80 ± 0.04), latency (horizontal CANVAS: 227 ± 38 ms, HC: 213 ± 25 ms; vertical, CANVAS: 249 ± 34 ms, HC: 235 ± 15 ms) and velocity in horizontal saccades (10°amplitude, CANVAS: 317 ± 60°/s, HC: 359 ± 62°/s; 15°amplitude, CANVAS: 406 ± 83°/s, HC: 452 ± 79°/s) (Fig. 1A–C).

**Fig. 2:**
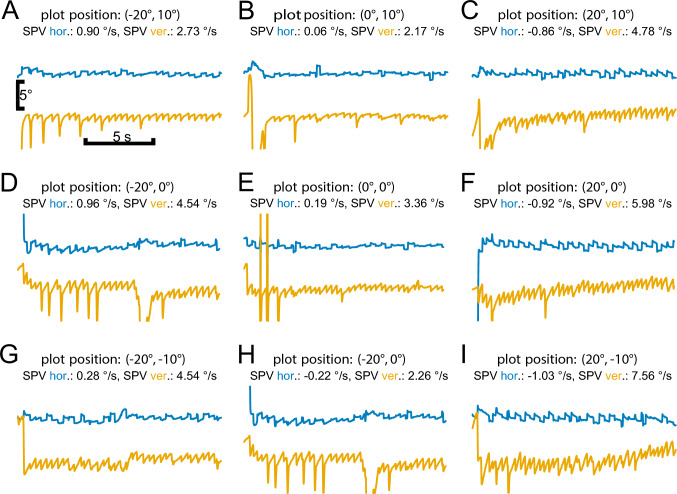
Gaze holding function: original gaze holding eye recordings during different target positions (**A**–**I**) in a patient. Horizontal and vertical target positions are displayed in brackets (positive amplitude equals right or up, negative amplitude equals left direction or down, respectively). Nystagmus is presented as mean SPV. Horizontal gaze-evoked (blue) and vertical down-beating (orange) nystagmus is present during all target positions. SPV increases on lateral and downward gaze. Abbreviation: hor. = horizontal, SPV = slow phase velocity, ver. = vertical

*Fixation and gaze holding*: Prolonged fixation revealed frequent square wave jerks in patients (*n* = 10) and occasionally in HC (*n* = 3). We neither found irrepressible saccades nor macrosaccadic oscillations. Spontaneous nystagmus was not found in any patient, but there was gaze-evoked nystagmus (GEN) during sustained eccentric vertical and horizontal fixation for at least 15 s in 10 patients (Table 2, Fig. 2A–I). Downbeat nystagmus (DBN) was found in 10 patients, with two patients in whom DBN changed into upbeating GEN in the supine position. After eccentric fixation (15 s), there was no rebound nystagmus. CANVAS patients without nystagmus were younger than patients with nystagmus (*U* = 4.0, *Z* = − 2.57, *p* = 0.008), with lower SARA scores (*U* = 8.0, *Z* =− 2.09, *p* = 0.040), but without different disease duration (*p* = 0.165).

**Table 2:** Descriptive statistics on the occurrence of horizontal and vertical nystagmus

	Central (only hor. nystagmus)	Left	Right	Central (only ver. nystagmus)	Up	Down
Number	7	10	10	10	10	10
Mean ± SD	− 0.31 ± 0.37	− 1.21 ± 0.90	1.69 ± 2.61	3.60 ± 3.09	2.98 ± 3.29	4.23 ± 4.22

Abbreviations: hor. = horizontal, SD = standard deviation, ver. = vertical

**Fig. 3:**
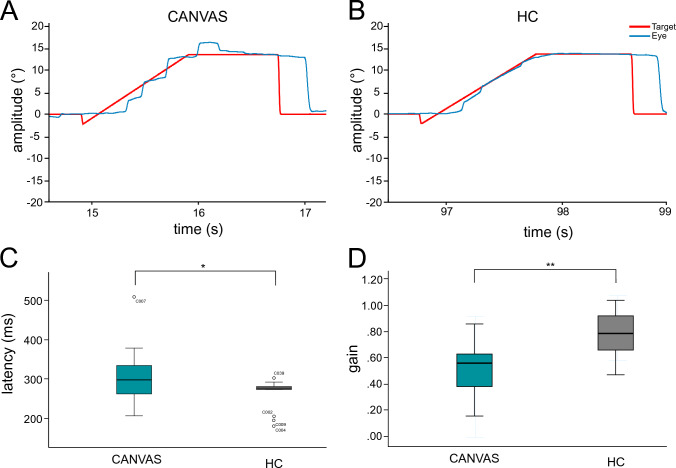
Smooth pursuit: Original step-ramp smooth pursuit eye recordings in a patient (**A**) and HC (**B**). Compared to HC, latency in patients is prolonged (**C**) with subsequently catch-up saccades leading to a reduced gain (**D**) in patients. * < 0.05, ** < 0.01. Abbreviation: CANVAS = cerebellar ataxia, neuropathy, vestibular areflexia syndrome, HC = healthy control

*Smooth pursuit*: Initiation of smooth pursuit onset in the step-ramp paradigm was delayed in patients compared to HC (CANVAS: 301 ± 75 ms, HC: 264 ± 39 ms; *U* = 59.0, *Z* = −2.008, *p* = 0.046, Fig. 3A–C). However, the initial pursuit acceleration was not different from HC (*p* = 0.290). The gain of sustained smooth pursuit was severely reduced (CANVAS: 0.53 ± 0.25, HC: 0.79 ± 0.18; *U* = 39.0, *Z* =− 3.035, *p* = 0.003, Fig. 3D). This was also seen in horizontal (CANVAS: 0.68 ± 0.19, HC: 0.89 ± 0.12; *U* = 39.0, *Z* =− 2.880, *p* = 0.003) and vertical (CANVAS: 0.60 ± 0.20, HC: 0.83 ± 0.18; *U* = 38.0, *Z* =− 3.076, *p* = 0.003) sinusoidal smooth pursuit (Supplementary Figure SF1A, B).

**Fig. 4:**
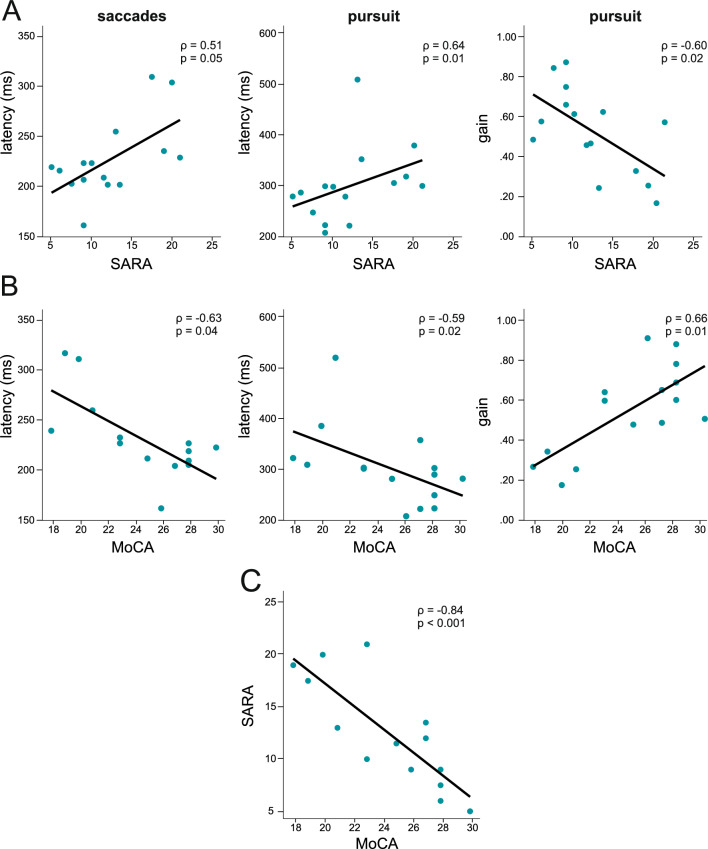
Correlations between SARA (**A**) or MoCA (**B**) scores with saccade and pursuit latency, pursuit gain and between themselves (**C**) in patients. Patients with prolonged latency or reduced pursuit gain performed poorer in clinical scores. In patients, high SARA scores occurred with lower MoCA scores. Abbreviations: MoCA = Montreal Cognitive Assessment, SARA = Scale of the Assessment and Rating of Ataxia

*Correlations*: None of the oculomotor parameters assessed in CANVAS patients correlated with disease duration, age at onset or VOR gain (*p* always > 0.241). Latency in reflexive saccades showed a positive trend with SARA scores (horizontal: *ρ* = 0.51, *p* = 0.050, vertical: *p* = 0.52, *p* = 0.050, Fig. 4A) and were prolonged with lower MoCA scores (horizontal: *ρ* =− 0.63, *p* = 0.011, vertical: *ρ* =− 0.53, *p* = 0.040, Fig. 4A). Additionally, latency in step-ramp smooth pursuit increased with larger SARA scores (*ρ* = 0.64, *p* = 0.010, Fig. 4B) and became larger with lower MoCA scores (*ρ* =− 0.59, *p* = 0.022, Fig. 4B). Impaired smooth pursuit gain was present with larger SARA scores (step-ramp: *ρ* =− 0.60, *p* = 0.018 [Fig. 4A], horizontal sinusoidal: *ρ* =− 0.74, *p* = 0.002, vertical sinusoidal: *ρ* = 0.70, *p* = 0.003) and lower MoCA scores (step-ramp: *ρ* = 0.66, *p* = 0.008 [Fig. 4B], horizontal sinusoidal: *ρ* = 0.66, *p* = 0.008, vertical sinusoidal: *ρ* = 0.64, *p* = 0.010). Increased SARA and low MoCA scores correlated with age (SARA: *ρ* = 0.65, *p* = 0.009; MoCA: *ρ* =− 0.72, *p* = 0.002) and with disease duration (SARA: *ρ* = 0.520, *p* = 0.047; MoCA: *ρ* =− 0.53, *p* = 0.040). SARA and MoCA also correlated negatively with each other (*ρ* =− −0.84, *p* < 0.001, Fig. 3C), i.e., SARA scores became larger with lower MoCA scores.

Apart from a positive correlation between vertical gain and MoCA score (*ρ* = 0.65, *p* = 0.030), none of the oculomotor parameters correlated with clinical scores in HC (*p* always > 0.082).

## Discussion

Based on clinical examinations, oculomotor abnormalities have been described in 79% of *RFC1* repeat expansion carriers [13]. Our goal was to quantitatively characterize the oculomotor phenotype of CANVAS beyond slow eye movements as clinical descriptions often deviate from quantitative recordings [12, 14]. Accordingly, quantitative eye movement data should be the basis for the classification of the oculomotor phenotype and for assessing the therapeutic efficacy, e.g., of aminopyridine. While Dupre et al. (2025) showed abnormal VVOR and slow eye movements in CANVAS patients [8], we provide recordings of fast eye movements in genetically confirmed CANVAS patients with cerebellar gaze holding deficits and abnormal initial pursuit responses, in the absence of saccade abnormalities.

Over 50% of patients showed GEN, many of them with omnidirectional nystagmus, 67% of them with a vertical velocity bias, almost exclusively DBN (92%). DBN was often small in amplitude baring the risk of being missed in the clinical examination [15]. This appears different from *FGF14* repeat expansion carriers (spinocerebellar ataxia type 27B, SCA27B) who frequently (70–85%, [2, 16, 17]) show DBN with larger amplitudes (clinical observation) although quantitative slow phase velocity values of recorded DBN have not published yet [18]. Gaze and head position dependency of our CANVAS patients was similar to patients with SCA27B: DBN increased with downward and lateral gaze as well as anteflexion of the head and decreased with upward gaze and retroflexion of the head. In a small portion (13%) of CANVAS patients in our study, DBN changed into vertical upbeating GEN and even upbeat nystagmus in the supine lying position. This gaze position dependency is usually caused by floccular impairment with an abnormal feedback loop to the brainstem neural integrator in cerebellar ataxias. This differs from structural paramedian medullary lesions involving the paramedian tract cells with inverse position dependency [19]. In contrast to SCA27B (100% [2]), we did neither identify rebound nystagmus nor cross-coupled nystagmus on head-shaking in our CANVAS patients.

*FGF14* repeat expansion carriers usually benefit from the treatment with fampridine (3,4 diamino- or 4-aminopyridine [4-AP]) with respect to the alleviation from oscillopsia, the functional impairment assessed by the Friedreich Ataxia Rating Scale and the reduction of slow phase velocity of DBN [16]. It has been suggested that the positive therapeutic response is indicative of *FGF14* repeat expansion carriers, in contrast to *RFC1* repeat expansion carriers (CANVAS patients). However, the efficacy of 4-AP on DBN in CANVAS patients has not been systematically studied yet. According to the gaze and gravity dependence of DBN in our CANVAS patients, 4-AP should be effective in a prospective study in reducing nystagmus amplitude [20]. However, therapeutic trials need to consider that high-resolution eye movement recordings are required as general cerebellar scores of functional restraints in daily life will not reflect improvement as bilateral vestibulopathy and polyneuropathy continue to impair postural control and gait. The degree of vestibular impairment and its progression clearly delineates *RFC1* (severe vestibulopathy) from *FGF14* (moderate—if any- vestibulopathy) repeat expansion carriers [2, 21].

Saccades in CANVAS patients, which had not been quantitatively studied before, were normal in our study: on average, there was no saccade dysmetria, normal velocity resulting in a normal amplitude/velocity relationship (main sequence). This contrasts previous clinical descriptions, which noticed saccade slowing in 19% of CANVAS patients [13] implicating extracerebellar, i.e., pontine neurodegeneration. Several hereditary cerebellar ataxias present with dysmetria of saccades, e.g., in SCA27B patients [2, 18] or spinocerebellar ataxia type 6 (SCA6) [22, 23]. Saccade latency and velocity were normal, which argues against potentially confounding cognitive impairment worsening oculomotor behavior. This is important since it has recently been shown that cognitive impairment is a common feature in CANVAS [4], which has been neglected in many studies so far. In keeping with this notion, our CANVAS cohort showed pathological MoCA values compared to HC. They had comparable scores (average: 24 points) to patients with mild cognitive impairment (MCI) and their saccade and pursuit latencies increased significantly with lower MOCA scores. Likewise, MCI patients usually show prolonged saccade latencies [24]. However, saccade latencies of our CANVAS patients were normal.

Cognitive decline with lower MOCA scores gets worse with longer disease duration of CANVAS, while there was no correlation with age in our healthy participants. In contrast, saccade and pursuit latencies did not correlate with disease duration. Cognitive impairment in CANVAS seems to develop with disease progression at a state when oculomotor abnormalities have already manifested. However, future studies should involve saccade paradigms that are more prone to cognitive decline in extracerebellar neurodegenerative diseases (e.g., antisaccades, memory-guided saccades).

Horizontal and vertical smooth pursuit eye movements of our CANVAS cohort were severely impaired, in line with clinical examinations [13] and recent recordings [8]. Clinically, this does not seem to delineate CANVAS from SCA27B [2, 17] or SCA6 patients [22].

Initial acceleration of smooth pursuit was not different from HC, but latency was prolonged in patients. Smooth pursuit initiation in the step paradigm requires rapid acceleration and thereby midline cerebellar functions [5]. Importantly, while unilateral posterior vermis and deep cerebellar nuclei lesions elicit direction-specific saccade hyper- and hypometria, bilateral lesions in chronic neurodegenerative diseases might functionally normalize pursuit acceleration [25]. Noteworthy, prolonged latency in pursuit initiation was probably not related to impaired attention, as this should have affected saccade latency as well.

From a neuroanatomic point-of-view, impaired smooth pursuit and DBN with GEN point to a midline cerebellar neurodegeneration, including the flocculus. The posterior vermis and the midline deep cerebellar nuclei usually cause saccade dysmetria in acute lesions and saccade hypometria in chronic lesions [25]. Our data suggest that pursuit pathways from the flocculus to the brainstem bypassing the midline cerebellar nuclei are preferentially involved in CANVAS.

Oculomotor abnormalities did not correlate with disease duration and the degree of vestibular hypofunction which is in line with the extracerebellar vestibular ganglionopathy in CANVAS. On the other hand, smooth performance became worse with larger functional impairment (SARA scores) and suggests that cerebellar involvement occurs late in the individual history of CANVAS. Longitudinal natural history studies are required to identify whether these oculomotor disorders occur after the onset of bilateral vestibulopathy and reflect cerebellar neurodegenerative progression in CANVAS.

From a clinical point of view, patients with slowly progressive gait ataxia are likely to suffer from CANVAS once bilateral vestibulopathy is severe as it delineates CANVAS patients from *FGF14* repeat expansion carriers (SCA27B) [2, 26] and patients with SCA6, apart from its pathognomonic chronic cough preceding gait ataxia for decades. In contrast to CANVAS, mild vestibulopathy in SCA27B probably reflects vestibulocerebellar neurodegeneration. If, however, cerebellar neurodegeneration precedes bilateral vestibulopathy it becomes much more difficult to identify CANVAS as similar cerebellar eye movement abnormalities are also found in SCA27B and SCA6.

## Conclusion

Smooth pursuit oculomotor abnormalities in our CANVAS patients are in line with other spinocerebellar ataxia syndromes (SCA27B, SCA6). Their intact visually guided saccades distinguish CANVAS patients from the other and reflect a midline cerebellar impairment without involvement of the brainstem. Mild cognitive impairment seems to be a feature of CANVAS and needs to be taken into consideration when latencies are used as progression markers. However, normal saccade function (latency, velocity, metria) argue against a clinically relevant attentional deficit. The correlation of SARA scores and disease duration together with the missing correlation between oculomotor abnormalities and disease duration and vestibulopathy indicates a late beginning of cerebellar involvement in CANVAS.

## Supplementary Information

Below is the link to the electronic supplementary material.

Supplementary file1 (DOCX 597 KB)

## Data Availability

Data are available from the corresponding author upon reasonable request.
