# Supplementary material for: Oculomotor abnormalities in patients with cerebellar ataxia, neuropathy, vestibular areflexia syndrome (CANVAS) reflect midline cerebellar impairment
Source: J Neurol. 2026 Feb 14;273(2):143. doi: 10.1007/s00415-026-13678-4 (PMC12906546; doi:10.1007/s00415-026-13678-4)
Supplement: Supplementary file 1 — Supplementary file1 (DOCX 597 KB) [file 415_2026_13678_MOESM1_ESM.docx]

# Supplementary Information

**Methods**

## Vestibular recordings

Quantitative analysis of the gain of the vestibulo-ocular reflex (VOR) by video-oculography of the horizontal head impulse test was performed as previously described [1, 5]. In brief, eye and head movements were recorded by a digital video camera (Eye-SeeCam HIT System, Autronics, Hamburg, Germany) at a sampling rate of 220 Hz. At least ten passive and rapid (peak velocity 250°/sec) head movements of small amplitude (10-15°) were performed per side. Head impulses were unpredictable for direction and amplitude. The gain of the horizontal vestibulo-ocular reflex was analyzed at a narrow time interval of 60 +/- 10 ms after head movement onset. A mean gain < 0.7 was considered pathologic. The mean VOR gain used in the present study was assessed by calculating the mean of the gain of the right and the left vestibular organ. The subjective visual vertical (SVV) was assessed with the head fixed on a chin rest by the subject’s adjustment of a bar to the perceived visual vertical without any spatial orientation clues in a dotted half-spherical dome, which is stationary or dynamic (moving visual background) around the line of sight. The normal range of SVV was defined as deviation of < ± 2.5°.

## Oculomotor recordings

**Apparatus:**

*Eyelink II system (SR Research Ltd., Ontario, CA; native sampling rate 500 Hz, resampled to 1000 Hz offline using a spline interpolation [6])*. During experiments, subjects sat in a comfortable chair; the head was immobilized by a chin rest and a forehead-holding device. The visual stimulus consisted of a red laser dot (diameter of 0.1°), rear-projected onto a translucent screen (Marata screen) at a viewing distance of 1.2 m. The laser dot was moved by two galvanometer scanners (GSI Lumonics, Munich, Germany), driven by an analog output card in the stimulus PC (AT-AO6/10, National Instruments, Munich, Germany). For calibration, we first presented a sequence of stimuli in central, horizontal, and vertical deflected saccadic and smooth pursuit positions. Subjects were asked to look at the laser dot as fast and accurately as possible.

*Video-based eyetracker (Eyelink 1000Plus, SR Research Ltd., Ontario, CA; Eyelink host software version 5.17; heuristic single-stage filter):* Stimuli were presented using a 27-inch LED monitor (XL2720, BenQ, Taipeh, Taiwan; 1920 x 1080 pixel, i.e., 49.3° x 28.9° visual size; 120 Hz refresh rate) and eye movements were recorded at 1000 Hz binocularly. Subjects sat in a comfortable chair and placed their head on a chin-rest and against a forehead-holding device. The stimulus consisted of a red dot (size 0.5°) on a black background moving on the screen at a viewing distance of 65 cm. System calibration included a built-in 13-step calibration and validation process. Additionally, we performed a 13-step calibration after recording eye positions at defined positions plus two sinusoidal smooth pursuit movements horizontally and vertically with an amplitude of +/-15° and a frequency of 0.2 Hz.

Recordings were performed in darkness for both systems. Visual acuity was normal. We recorded both eyes but only movements of one eye (by default the left eye) were analyzed. Pre-processing of the data was performed by transformation of data from pixel to degrees of visual angle with no additional filtering. Eye velocity for sinusoidal smooth pursuit paradigms was computed using a 5-point central median difference algorithm and a 70 Hz Gaussian filter subsequently. For step-ramp paradigms a 9-point central median difference algorithm was used with a 30 Hz Gaussian filter.

**Oculomotor tasks:**

**Saccades**: Gain, latency and velocity of pro-saccades to visual targets at different locations and displacements (10°, 15°) were examined for the horizontal and vertical direction (30 trials per direction). Direction and amplitude were randomized, the preceding fixation phase was varied from 1000 to 1500 ms followed by a gap of 200 ms at the onset of lateral target step. The lateral target position varied between 1800 and 2300 ms. Latency was the interval from stimulus to saccade onset. Only latencies above 120 ms and below three times the standard deviation were included to rule out express or prolonged latencies. Eye velocity was calculated as the difference of median eye position of five data points before and after the actual data point] * sampling rate (1000 Hz).

**Gaze-holding function:** Fixation nystagmus was tested with eyes open during fixation in 9 different positions: centered (0°-amplitude horizontally and vertically), lateral (20° horizontal amplitude to the left or right direction), up or down (10° vertical amplitude) and lateral and up/down combination (e.g., 20° horizontal amplitude to the right and 10° vertical amplitude up). Each position was held for 15 s with a 200 ms gap in between.

**Smooth pursuit paradigms:** Predictive, closed-loop smooth pursuit was tested in a sinusoidal smooth pursuit paradigm composed of horizontal and vertical oscillations of 0.2 Hz. For the Eyelink II system, the stimulus consisted of amplitudes of 15.9°; i.e., maximum stimulus velocity of 20°/s with 10 cycles per direction in total. For the Eyelink 1000Plus recoding system, the use of the same vertical stimulus was not possible due to the monitor’s limitations. Therefore, the vertical amplitude was 12° with a maximum stimulus velocity of 19.9°/s with 20 cycles in total. After elimination of saccades, the phase and amplitude of a sinusoid were adjusted to match the slow phase velocity of the eye. The fitting was performed with the least-squares method.

The step-ramp paradigm [2, 7] was used as described before [3, 8] to quantify the initial response of unpredictive smooth pursuit without visual feedback (open loop) and the closed-loop period. During each trial, the target stepped away horizontally from the gaze straight ahead position (variation of 1750 ± 250 ms) and then moved with a constant velocity in the opposite direction. Because we used step amplitudes of 2.4° and ramp velocities of 16 °/s, the stimulus passed the center after 150 ms, thus allowing smooth pursuit initiation without an initial saccade. It continued lateral for 1000 ms and stayed at its final position for 750 ms ± 250 ms. Each sequence consisted of 20 ramps each to either side in random order. Foveofugal ramps (4 to the left and 4 to the right) with horizontal target steps away from the center position and consecutive constant velocity stimuli in the same direction were interspersed to keep the level of attention high. The duration of the fixation interval before each trial was varied from 1600 to 1900 ms randomly [3].

Analysis of pursuit acceleration: The onset of pursuit acceleration (pursuit latency) was defined as the time when eye velocity exceeded 3.2 times the standard deviation of baseline velocity signal (measured over a 200 ms interval before the target started to move). Subsequent data in a 60 ms time window were used to calculate the slope of a least square fit (robustfit function within Matlab®) of eye velocity [2], as described in details before [4].

## Data and statistical analysis

Our data was collected from two different eye movement recording systems (magnetic scleral search-coil system, Eyelink II) with different target projections (laser projection on wall vs. pixel on monitor) leading to differences regarding latency and initial acceleration in saccades and saccade tasks (eye amplitude/target amplitude). Therefore, we performed separate z-transformation for both recording systems to compare results between groups system-independent. For better understanding, results of latency and initial acceleration were recalculated back into their standard units (ms and °/ms²) and stated as such.

**Results**

## Smooth pursuit

**Figure 1:** Horizontal (**A**) and vertical (**B**) sinusoidal pursuit gain in patients (blue) and HC (grey). Patients performed sinusoidal smooth pursuit less accurate than HC for both directions. ** < 0.01. Abbreviations: CANVAS = cerebellar ataxia, neuropathy, vestibular areflexia syndrome, HC = healthy control subjects, hor. = horizontal, ver. = vertical.

References

1. Borsche M, Tadic V, König IR, Lohmann K, Helmchen C, Brüggemann N (2022) Head impulse testing in bilateral vestibulopathy in patients with genetically defined CANVAS. Brain and Behavior 12:e32546

2. Carl JR, Gellman RS (1987) Human smooth pursuit: stimulus-dependent responses. JNeurophysiol 57:1446-1463

3. Hubner J, Sprenger A, Klein C, Hagenah J, Rambold H, Zuhlke C, Kompf D, Rolfs A, Kimmig H, Helmchen C (2007) Eye movement abnormalities in spinocerebellar ataxia type 17 (SCA17). Neurology 69:1160-1168 10.1212/01.wnl.0000276958.91986.89

4. Lencer R, Sprenger A, Trillenberg P (2019) Smooth Eye Movements in Humans: Smooth Pursuit, Optokinetic Nystagmus and Vestibular Ocular Reflex. In: Klein C, Ettinger U (eds) Eye Movement Research. An Introduction to its Scientific Foundations and Applications. Springer, pp 120-154

5. Machner B, Erber K, Choi JH, Sprenger A, Helmchen C, Trillenberg P (2021) A simple gain-based evaluation of the video head impulse test reliably detects normal vestibulo-ocular reflex indicative of stroke in patients with acute vestibular syndrome. Frontiers in Neurology 12:741859

6. Mack DJ, Belfanti S, Schwarz U (2017) The effect of sampling rate and lowpass filters on saccades–a modeling approach. Behavior research methods 49:2146-2162

7. Rashbass C (1961) The relationship between saccadic and smooth pursuit tracking eye movements. JPhysiol 159:326-338

8. Sprenger A, Hanssen H, Hagedorn I, Prasuhn J, Rosales RL, Jamora RDG, Diesta CC, Domingo A, Klein C, Bruggemann N, Helmchen C (2019) Eye movement deficits in X-linked dystonia-parkinsonism are related to striatal degeneration. Parkinsonism Relat Disord 61:170-178 10.1016/j.parkreldis.2018.10.016
